# In the jungle of time: the concept of identity as a way out

**DOI:** 10.3389/fpsyg.2014.00844

**Published:** 2014-07-29

**Authors:** Bin Zhou, Ernst Pöppel, Yan Bao

**Affiliations:** ^1^Institute of Psychology, Chinese Academy of SciencesBeijing, China; ^2^Department of Psychology, Peking UniversityBeijing, China; ^3^Human Science Center, Ludwig-Maximilians UniversityMunich, Germany; ^4^Institute of Medical Psychology, Ludwig-Maximilians UniversityMunich, Germany; ^5^Parmenides Center for Art and SciencePullach, Germany; ^6^Key Laboratory of Machine Perception (MoE), Peking UniversityBeijing, China

**Keywords:** time, identity, homeostasis, circadian rhythm, subjective present, simultaneity, episodic memory

## Abstract

What could be a unifying principle for the manifold of temporal experiences: the simultaneity or temporal order of events, the subjective present, the duration of experiences, or the impression of a continuity of time? Furthermore, we time travel to the past visiting in imagination previous experiences in episodic memory, and we also time travel to the future anticipating actions or plans. For such time traveling we divide time into three domains: past, present, and future. What could be an escape out of this “jungle of time” characterized by many different perceptual and conceptual phenomena? The key concept we want to propose is “identity” which is derived from homeostasis as a fundamental biological principle. Within this conceptual frame two modes of identity are distinguished: individual or self-identity required because of homeostatic demands, and object-related identity necessary for the reliability and efficiency of neuro-cognitive processing. With this concept of self- and object-identity, the different temporal experiences can be conceptualized within a common frame. Thus, we propose a fundamental biological principle to conceptually unify temporal phenomena on the psychological level.

## DIVERSITY OF TEMPORAL EXPERIENCES AND CONCEPTS

When thinking about the different temporal experiences one gets the impression that one is dealing with very different topics which are not related to each other (Pöppel, 1978; Pöppel and Bao, 2014); temporal experiences appear to be qualitatively distinct. The question that has to be addressed first, is, what is meant with “experience”: do we have “temporal experiences” at all, or are we not usually referring to theoretical concepts? We take the position that we often confuse experiences as “direct experiences” (in German “Erleben”) and abstract notions (in German “Erfahrung”), without being aware of a categorical error; the English term “experience” apparently does not differentiate between these two meanings. We should remember what Gibson once said (Gibson, 1973): “Events are perceivable but time is not.” And in a similar way Woodrow (1951) states: “Time is not a thing that, like an apple, may be perceived.”

What are some of the problems that make it difficult to find a unifying principle of temporal experiences? A good example is the one when we refer to the “present.” Do we think of the present as a border with no temporal extension between past and future, or does the present have a temporal extension? If the present is just a border without temporal extension between past and future, it cannot have any experiential quality. In this case the present is a theoretical notion, and temporal experiences are reduced on the phenomenal level to what has happened and what might happen. But what could be the experiential quality of what is going to happen or what has happened? Temporal experiences are then either anticipations, hopes, plans or memories of events past. But if the present has a temporal extension, as is implicitly assumed by Augustinus (397/8, 1993), one of the founding fathers of “temporal philosophy,” or James (1890), then the empirical question comes up how long such a present might be, and this question has indeed been addressed with different experimental paradigms (Pöppel, 1978; Pöppel and Bao, 2014; Wittmann, 2014). And then the question comes up: does this temporal interval of finite duration move continuously along the arrow of time, or does it move in discrete steps along an abstract temporal axis?

There is another problem which is usually neglected: does the English word “present” cover equivalent connotations in Chinese, German or any other language? Can we disregard the language we are using in such discourses? Cultural neuroscience is dealing exactly with this problem of cultural specifics contrasting them with anthropological universals (Bao and Pöppel, 2012). Thus, we might even move around in a conceptual jungle defined by different languages, in which specific terms only superficially appear to be equivalent. The English “present” evokes different associations compared to the German “Gegenwart” or the Chinese “xian zai.” “Present” is associated with sensory re*present*ations, whereas “Gegenwart” has a more active flavor; the component “warten” refers either to “take care of something” or “to wait for something,” and it is thus also past and future oriented. “Xian zai” is associated with the experience of existence in which something is accessible by its perceptual identity, it implies a spatial reference indicating the “here” as the locus of experience, and it is also action oriented. Although the different semantic connections are usually not thought of explicitly, they still may create a bias within an implicit frame of reference (Pöppel and Bao, 2011).

Being already in the jungle of time with reference to the present only, the jungle gets deeper if further temporal phenomena are included. Is the experience of simultaneity as obvious as it appears at first sight? Simultaneity as a perceptual experience is for instance indicated by the philosopher Kant (1781/1787) in his “Critique of Pure Reason” when he analyses time as an *a priori* form of knowledge. We claim that there is nothing like such an experience. It would require that (at least) two independent experiences on a perceptual or conscious level at exactly the same time take place; this we consider as impossible. On the basis of a retrospective evaluation of perceived events we may conclude that they have been simultaneous; on this basis of reasoning, simultaneity is a theoretical construct with no experiential quality.

The conclusion that temporal experiences are often retrospectively constructed and not representing “experiences” at all, can also be drawn from experiments measuring temporal order threshold (Bao et al., 2013a, 2014a). If for auditory stimuli their temporal sequence has to be indicated, subjects may use a holistic strategy by perceptually fusing the two sequential stimuli with different frequency into one percept with tones going up or going down; on the basis of the direction of the perceptual movement, subjects reconstruct the sequence of events retrospectively. The same applies to the case when one refers to spoken Chinese as a tonal language: the second, third and fourth tones are characterized by frequency changes with immediate experiential quality defining the meaning of a word within a semantic frame; only retrospectively and on an abstract level the tones may be defined as auditory stimuli with typical changes of frequency and the changing order, but in the moment of hearing they are not distinguished on an analytical level.

There are more temporal phenomena which appear to be unrelated to others: why do experiences with the same objective duration may have different subjective durations? What happens when we are bored in which case our attention is drawn to the passage of time and time appears to slow down? But is it “time” that slows down? One can argue that attention is drawn to a reduced availability of information, and that only secondarily this mental state is interpreted as a slowing down of time itself. Thus, one is again confronted with a potential confusion of categories, one being experiential and the other being conceptual. Furthermore, what gives us the impression of a continuity of time (Pöppel, 2009)? Also in this case we have to address the question whether we deal with an experiential quality of continuous time, or whether we derive a concept of temporal continuity on the basis of what is represented in consciousness.

To overcome the apparent diversity of temporal experiences or concepts, time as defined in classical physics might provide a unifying principle; Newton writes (Newton, 1686): “Absolute, true, and mathematical time, of itself, and from its own nature, flows equably without relation to anything external.” In this definition time is considered to be a medium or one-dimensional “container” within which temporal experiences are implemented. Such a mapping of subjective time onto Newtonian time has been dominant in psychological research (e.g., Pöppel, 1978); but then one is confronted with another problem: in modern physics one has to deal with different concepts of time as formulated for instance in the theories of relativity, cosmology, the second law of thermodynamics or dissipative structures (Ruhnau, 1994). Why should one select the classical concept of time as a unifying principle if others and more advanced ones exist? Furthermore, it is important to stress that there is no concept of a “now” in physics (Ruhnau and Pöppel, 1991); thus, a useful reference for a better understanding of psychological phenomena does not exist in physics.

Moving to another path in this jungle of time makes it even more unlikely to find a solution in physics: we can mentally travel to the past visiting previous experiences in our episodic memory; when we do so we discover that our pictorial memories are always related to specific places, that they have been imprinted by a strong emotion, and that we are pictorially confronted with ourselves in these images becoming our own doppelganger. Thus, these internal images are no longer copies of objective events from the past, because in reality we are physically never present in an image we have in front of our eyes.

There are even more temporal phenomena which have to be dealt with on the psychological level if one is looking for a unifying principle. Human behavior is embedded within geophysical cycles like the diurnal or annual rhythms which give rise to the experience of a day or a year which defines a subjective and objective frame for the temporal organization of life. These rhythms are controlled by biological clocks (Aschoff, 1965; Roenneberg and Aschoff, 1990), and they carry an evolutionary heritage in our “temporal genes.” They are responsible for feeling embedded in the temporal structure of the natural environment.

## IDENTITY AS NECESSARY CONDITION FOR HOMEOSTASIS IN TIME

What could be an escape out of this jungle of diverse temporal phenomena? What might be a unifying principle that conceptually binds these phenomena together? We suggest leaving behind “time” as a conceptual frame for such an integrating enterprise. Instead, we want to propose as a unifying frame the concept of “identity” (Pöppel, 2010), i.e., the defining characteristics by which a thing or person is recognized as a persisting entity over time. The argument is based on the biological principles of homeostasis (Bernard, 1856/1957; Gross, 1998) and allostasis (Sterling, 2004) and their consequences for behavioral control. Homeostasis as originally suggested by Bernard (1856/1957) is essential for all biological processes; physiological systems like the regulation of body temperature in homoiothermic organisms have to be kept stable to guarantee the maintenance of life. Allostasis (Sterling, 2004) expands this concept by stressing the anticipative and adaptive regulation of bodily functions which unfold over time; bodies are designed for efficiency, and the prediction of future states is required. A similar concept of including future states has been suggested in a generalization of the reafference principle (von Holst and Mittelstaedt, 1950; Bao et al., 2014b); efference copies are compared with sensory reafferences to allow a continuous self-monitoring of behavior and anticipate future environmental constellations.

Taking this biological perspective every organism including every human being has to establish and maintain a homeostatic state throughout time. This necessarily implies a “self” (Pöppel, 2010), i.e., the identity of the organism to achieve this individual goal. As the homeostatic state may be violated because of perturbations by unexpected stimuli or changes of the metabolic state expressed for instance in the feeling of hunger, thirst or sexual desire, the organism constantly monitors its internal state in order to maintain homeostasis. What are the operating mechanisms for these activities to keep equilibrium? The answer to this question is based on a taxonomy of functions (Pöppel, 1989) which distinguishes between content and logistical functions; temporal processing represents a logistical function, whereas percepts or memories refer to content functions.

What an organism has to do for the creation and maintenance of homeostasis is to determine a functional state of the organism using sensory systems. This requires a time interval of finite duration within which sensory information is integrated. The challenges for the brain in creating identity become obvious if one considers the neuronal machinery for perceptual processing. Taking visual and auditory perception, one has to deal with the problem that the time required for the transduction of optic and acoustic information in the two modalities is different, being much shorter in the auditory modality (Pöppel et al., 1990). Furthermore, transduction in the retina is dependent on flux such that the necessary information to define a visual object with different areas of brightness arrives at different times in cortical areas. Thus, to create for instance the perceptual identity of another person whom we see and who talks, our brain has to overcome temporal uncertainty of the neuronal information. It is suggested that this logistical problem is solved by the use of neuronal oscillations (Pöppel, 2009; Pöppel and Bao, 2014). Stimulus-entrained oscillations with a period of some tens of milliseconds are used to integrate the neuronal information. Experimental evidence indicates that information within such temporal window is a-temporal in nature; the before and after relationship of events can not be extracted within such intervals. All information within one period of the oscillation is treated as co-temporal, thus, allowing the definition of a functional state. (This neuronal operation also implies that the brain steps out of a continuity of time as defined in Newtonian physics.)

Support for this notion comes from experiments on temporal order threshold in different sense modalities (Hirsh and Sherrick, 1961; Bao et al., 2013a, 2014a); only if some tens of milliseconds have passed, it is possible to indicate their correct sequence. Evidence for such a temporal mechanism is also provided by measurements of choice reaction time or pursuit eye movements (Harter and White, 1968; Pöppel, 1970, 1972; Pöppel and Logothetis, 1986); response histograms show multimodalities with modal distances of 30–40 ms, which are explained by an underlying process of discrete time sampling. These observations implying stimulus-triggered neuronal oscillations have also been made visible in studies on auditory evoked potentials (Galambos et al., 1981; Madler and Pöppel, 1987; Schwender et al., 1994).

To program a successful movement trajectory toward an anticipated goal in order to maintain or reach homeostasis, at least two such functional states have to be defined for reasons of comparison. A difference between functional states indicating different physical or psychological distances toward the goal suggests the direction of a movement trajectory, i.e., an action or more generally speaking a plan. If no difference is determined, no action is required. The necessary comparison between functional states is only possible if the distinct states keep their identity. On the basis of a comparison, a choice and a decision can be made for an action that is characterized by anticipating the consequences of such a decision. Anticipation implies the maintained identity of both actor and the goal of action. Thus, all sequential operations require the identity of functional states, the results of the comparisons, the content of the decisions, and the anticipated goals to be reached. If temporal processing is distorted, as can be observed in certain brain diseases (Teixeira et al., 2013), the consistency and coherence of our mental life may break down; pathological changes which may occur at different levels of temporal processing support the outlined mechanisms of cognitive control.

As suggested in a model of hierarchical temporal processing (Pöppel, 1997), a comparison between successive functional states has to happen within a “temporal window” (Pöppel and Bao, 2014) of a few seconds; if the delay between the states to be compared is too long, the representation of the first one may have faded away. We suggest that the “subjective present” in humans with the duration of a few seconds serves the purpose of meaningful comparisons. Experimental evidence suggests that a specific neuronal mechanism creates a temporal window with the duration of ∼3 s. A pre-semantic temporal integration mechanism provides a temporal stage on which conscious activity is represented. For every time window of up to 3 s, the identity of what we perceive, what we remember or what we think of is maintained.

Examples for such a temporal integration within a few seconds providing perceptual identity of what is represented come from the duration of intentional acts in humans (Schleidt et al., 1987; Nagy, 2011) and other higher mammals (Gerstner and Fazio, 1995), sensorimotor synchronization (Mates et al., 1994), and spontaneous speech (Vollrath et al., 1992), as well as observations of disrupted temporal integration in patients having suffered speech deficits (Szelag et al., 1997) or in autistic children (Szelag et al., 2004). Most importantly, this temporal window of some 3 s is also used to temporally integrate spatial attention. Using the paradigm of inhibition of return (IOR) it has been discovered that the perifoveal region of the visual field is characterized by a different attentional control mechanisn compared to the periphery (Bao and Pöppel, 2007); although the decay functions of IOR are different for the two attentional fields, they share the same time window (Bao et al., 2013b).

With the hierarchically connected neurocognitive machineries to define the temporal order and an integration interval for optimal comparisons (Pöppel, 1997), the brain also owns a mechanism to create experiences of what we interpret as different subjective durations. If in a pre-semantically defined temporal window of a few seconds (Pöppel, 2009) more or less information is integrated on the basis of defined functional states, subjective durations are retrospectively appreciated as having been long or short. However, to refer to such different durations is only possible if the functional states maintain their identity within the temporal window of 2–3 s. The derived impression of continuity of time on a presumably higher level of processing requires the semantic connection of what is represented in successive temporal windows which again entails the identity of such successive contents.

The circadian clock (Merrow et al., 2005) creates a particular challenge with respect to individual identity as different physiological and psychological functions show different diurnal patterns (e.g., Lotze et al., 1999), and the same can be suspected for circannual cycles. The “phase-map” is such that an identity of the constellation of all psychological and physiological functions is only observed in intervals of 24 h; we are not “the same” throughout a day and possibly also throughout the year, and everybody returns to his or her psychophysiological self only at regular intervals defined by geophysical cycles. However, these regular fluctuations are usually masked and we can refer to our self effortlessly (Han and Northoff, 2009; Pöppel and Bao, 2011; Zaytseva et al., 2014). The creation of individual identity over time is made possible by functional memory systems operating both on an implicit and explicit level (Pöppel and Bao, 2011). The importance of memory systems for the creation of individual identity is documented also by time travels to the past when we visit images in our episodic memory. As indicated above, one is confronted in such time travels with the representation of oneself, with the own doppelganger, and it is suggested that this doubling of oneself is essential for the construction of personal identity: we double our self and in doing so we can refer to our self. Taken together, we suggest that the concept of identity provides a common frame for different temporal phenomena and, thus, allow an escape out of “the jungle of time.”

## Conflict of Interest Statement

The authors declare that the research was conducted in the absence of any commercial or financial relationships that could be construed as a potential conflict of interest.
